# Using Multi-Instance Hierarchical Clustering Learning System to Predict Yeast Gene Function

**DOI:** 10.1371/journal.pone.0090962

**Published:** 2014-03-12

**Authors:** Bo Liao, Yun Li, Yan Jiang, Lijun Cai

**Affiliations:** College of Information Science and Engineering, Hunan University, Changsha, Hunan, China; University of Bonn, Bonn-Aachen International Center for IT, Germany

## Abstract

Time-course gene expression datasets, which record continuous biological processes of genes, have recently been used to predict gene function. However, only few positive genes can be obtained from annotation databases, such as gene ontology (GO). To obtain more useful information and effectively predict gene function, gene annotations are clustered together to form a learnable and effective learning system. In this paper, we propose a novel multi-instance hierarchical clustering (MIHC) method to establish a learning system by clustering GO and compare this method with other learning system establishment methods. Multi-label support vector machine classifier and multi-label K-nearest neighbor classifier are used to verify these methods in four yeast time-course gene expression datasets. The MIHC method shows good performance, which serves as a guide to annotators or refines the annotation in detail.

## Introduction

Genes are annotated in gene annotation databases [e.g., gene ontology (GO), KEGG, and MIPS], but the rate of gene identification is faster than gene annotation. Given that large amounts of identified genes, predicting the functions for un-annotated genes is a challenge. To date, many effective machine learning techniques are proposed. However, function prediction is different from the common machine learning tasks. A gene may have multiple functions and the function belongs to a set of genes. Function prediction belongs to the multi-label learning (MLL) task, and the common machine learning task is a single-instance single-label learning. Therefore, establishing an effective and learnable learning system for learning machines is necessary.

In this study, different types of data have different learning approaches. We choose yeast time-course gene expression datasets because they record gene responses to various environments. Therefore, when searching for functions of a gene according to their involvement in biological processes, measurements of changes in gene expression throughout the time course of a given biological response are particularly interesting [1].

Gene function prediction method for different purposes can be grouped into supervised and unsupervised methods. Unsupervised methods (i.e., clustering) do not usually use existing biological knowledge to find gene expression patterns. Eisen et al. [2] discovered classes of expression patterns and identified groups of genes that are regulated similarly. Ernst et al. [3], [4] clustered short time series gene expression data using a predefined expression model. Ma et al. [5] used a data-driven method to cluster time-course gene expression data. Other popular clustering algorithms include hierarchical clustering (HC), K-means clustering, and self-organizing maps [6]. Supervised methods (i.e., classification) use existing biological knowledge, such as GO, to create classification models. Lagreid et al. [1] applied the If-Then Rule Model to recognize the biological process from gene expression patterns. GENEFAS [7] predicted functions of un-annotated yeast genes using a functional association network based on annotated genes. Clare [8] presented a hierarchical multi-label classification (HMC) decision tree method to predict *Saccharomyces cerevisiae* gene functions. Schietgat et al. [9] presented an ensemble method (i.e., CLUS-HMC-ENS), which learns multi-tree for predicting gene functions of yeast. Kim et al. [10] combined the predictions of functional networks with predictions from a Naive Bayes classifier. Vazquez et al. [11] predicted global protein function from protein–protein interaction networks. Deng et al. [12] predicted gene functions with Markov random fields using protein interaction data. Nabieva et al. [13] proposed the functional flow method, which is a network-flow based algorithm, to predict protein function with few annotated neighbors. Recently, Magi et al. [14] annotated gene products using weighted functional networks. Liang et al. [15] predicted protein function using overlapping protein networks. Mitsakakis et al. [16] predicted *Drosophila melanogaster* gene function using the support vector machines (SVMs).

The present study predicts gene function based on the assumption that genes participating in the same biological processes have similar expression profiles. We initially produce a non-noise system by selecting genes. Then, the multi-instance hierarchical clustering (MIHC) method is proposed to establish a learning system. Finally, multi-label support vector machine (MLSVM) and multi-label K-nearest neighbor (MLKNN) classifiers are used to predict the function of genes in time-course expression profile. The experiment proves the feasibility and efficiency of the proposed method.

## Materials and Methods

### Gene function prediction

In the GO database, the GO terms are organized as a directed acyclic graph (DAG). In the GO hierarchical structure, the genes are annotated at various levels of abstraction. When genes are annotated with the GO terms, the genes are annotated with the highest possible level of details, which corresponds to the lowest level of abstraction [17]. Therefore, the goal of gene function prediction is for the annotators to annotate genes with the highest level GO terms. However, we can only obtain extremely few positive genes with similar GO terms, and little information is available for a machine learning system. To obtain more positive genes and efficiently predict gene function, many researchers up-propagate gene annotation along a GO hierarchical structure and establish a learning system. The up-propagation approach can substantially group the following two methods: cluster genes to a certain GO level [8], [9] and to a certain number [18].

### Multi-instance learning (MIL) and MLL

Zhou et al. [19] provided a detailed description on MIL and MLL. MIL and MLL are used to learn the function of 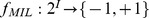 from the datasets 
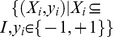
 and 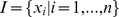, and 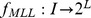 from the datasets 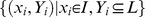 and 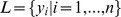, respectively.

**Figure 1:**
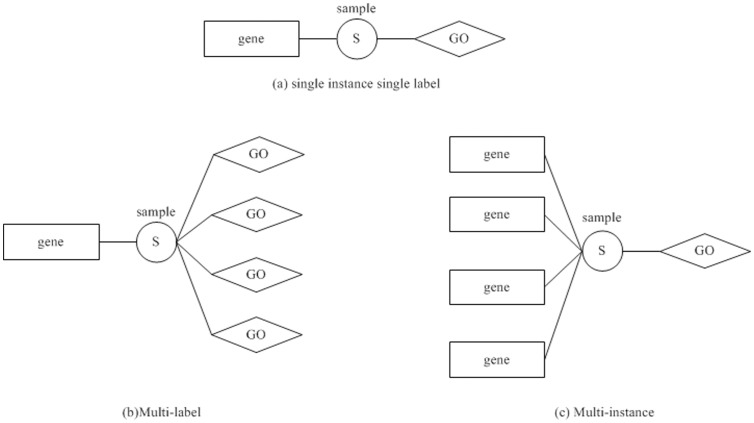
Three types of learning task. (a) A gene is treated as a sample and owns one GO term only, which is called the single instance single label. (b) A gene is treated as a sample and annotated by multiple GO terms. This relationship between gene and GO terms is multi-label. (c) Multiple genes are treated as samples and share the same GO term. The relationship between genes and GO term is called multi-instance.

The relationships between genes and annotations are found in the GO database (Figure 1). Figure 1(b) shows that a gene is annotated by multiple GO terms, and Figure 1(c) shows that the genes are treated as instances of the sample with the annotation of GO. This GO term can be represented by those genes. Therefore, the relationships between gene and GO shown in Figures 1(b) and 1(c) are called multi-label and multi-instance, respectively.

### MLSVM

SVM is an effective machine learning method. For classification problems, SVM implements a large margin classifier by solving a quadratic optimization program on the basis of the principle of structural risk minimization. Li et al. [20] adjusted the SVM to multi-label classification by improving the quadratic optimization program. Suppose 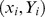 is a training sample, where 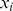 is the feature vector and 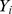 is the sample label. Let 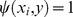 if 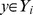 and 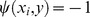, otherwise the SVM classification problem model is described by the following optimization problem:
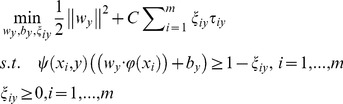
(1)where 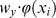 is the inner product, 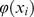 is the function that maps 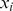 to a higher dimensional space 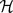, 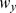 and 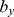 are the parameters for representing a linear discriminant function in 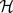, 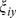 is the non-negative slack variable introduced in the constraints to permit some training samples to be misclassified, 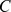 is the parameter to trade off the model complexity, and 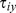 is the amplification coefficient of the loss 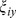 for handing the class imbalance problem [20], [21].

Compared with the model proposed by Vapnik [22], the aforementioned model performs better in multi-label classification. Generally, multi-label classification is transformed to multiple binary classifications. Class imbalance problem is a considerable barrier for each binary classification. The parameter 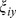 in Eq. (1) addresses this problem with a good performance.

### MLKNN

The K-nearest neighbor is another stable popular machine learning method. This method performs more rapid classification than the SVM. Zhang et al. [23] improve KNN method for multi-label classification, which served as our motivation in our proposed model. In the MLKNN model, the candidate classes of a given test sample 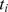 are obtained by
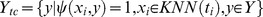
(2)where 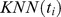 is the k-nearest neighbor of 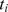 among the training set 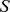. For each candidate class 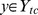, the following likelihood score is calculated
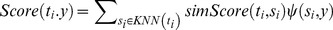
(3)where 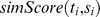 is the similarity score of 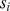 to 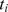. The labels of 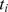 are calculated by
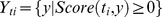
(4)


### Gene selection

We are not interested in all genes in the gene expression profiles. In gene function prediction, we assume that genes participating in the same biological processes have similar expression profiles [2], [24]. For this proposal, we select genes that are significantly correlated with each other in the same function. Let 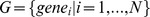, 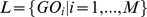, and 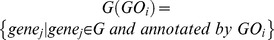, where 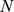 is the number of genes, and 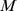 is the number of GO terms. For each 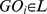, we draw a graph 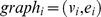 for genes that significantly correlate with each other. 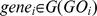 represents the 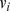 of 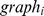. An edge exists between 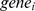 and 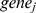 if 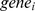 and 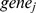 are significantly correlated. We define 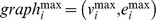, which is the maximum clique of 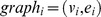 and 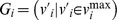. However, the maximum clique problem is complete NP-hard [25]–[27]. In this paper, a greedy algorithm is used to deal with this problem, and the non-noise system of expression data and annotation are represented as 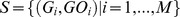.

### Learning system establishment method

**Figure 2:**
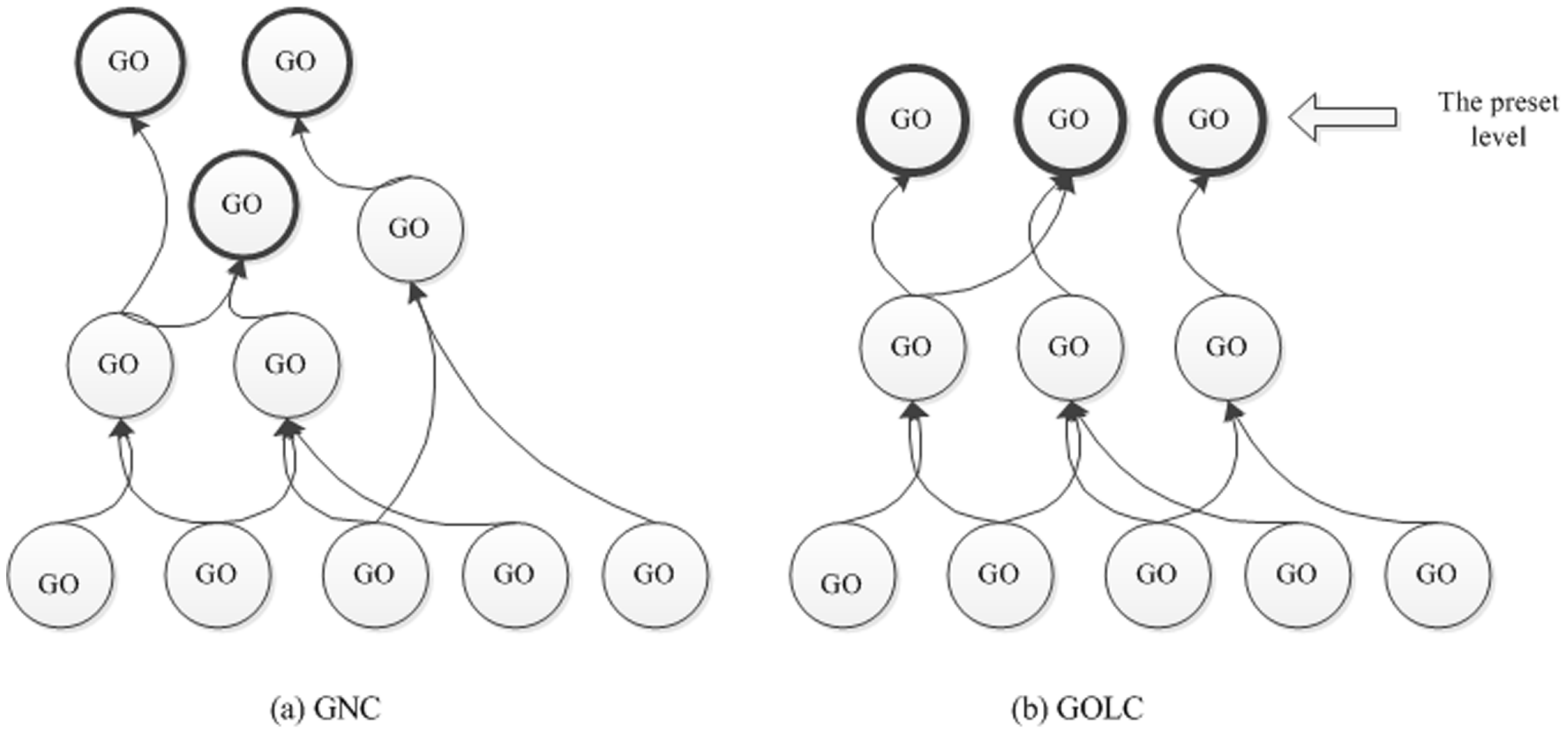
GO in the last level up-propagate along GO DAG. (a) The bold GO terms all own at least λ genes. (b) The bold GO terms are in the 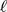 level of GO DAG.

Prior to the prediction of gene function, we establish a learning system for classification. Learning system establishment is the reconstitution of gene labels. GO DAG and MIPS are usually used to aid the establishment of learning systems. Clare et al. [8] and Schietgat et al. [9] established an MIPS-based learning system. Based on GO DAG, we use the same approach as those in [8] and [9]. We called this method GO level clustering (GOLC), which up-propagates the gene annotations to a preset GO level 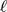, such as the first level (i.e., 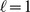) of the GO DAG, and cluster genes. In another approach, Hvidsten et al. [18] used the method called gene number clustering (GNC) to establish the learning system. The GNC method let the annotations up-propagate along the GO DAG until each annotation has at least 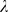 genes (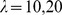 in [18]). Figure 2 shows the two aforementioned methods.

### MIHC method

The HC method is a widely used machine learning technology in the clustering algorithm. Johnson [28] proposed the extensively studied hierarchical clustering scheme (HCS). The HCS initializes all sample dissimilarities and then forms a cluster from the two closest samples or clusters. These steps are repeated until all samples are clustered to one group. Therefore, we can set a terminal factor to stop the cluster rather than preset the number of groups. Thus, HCS is suitable for all kinds of datasets.

**Figure 3:**
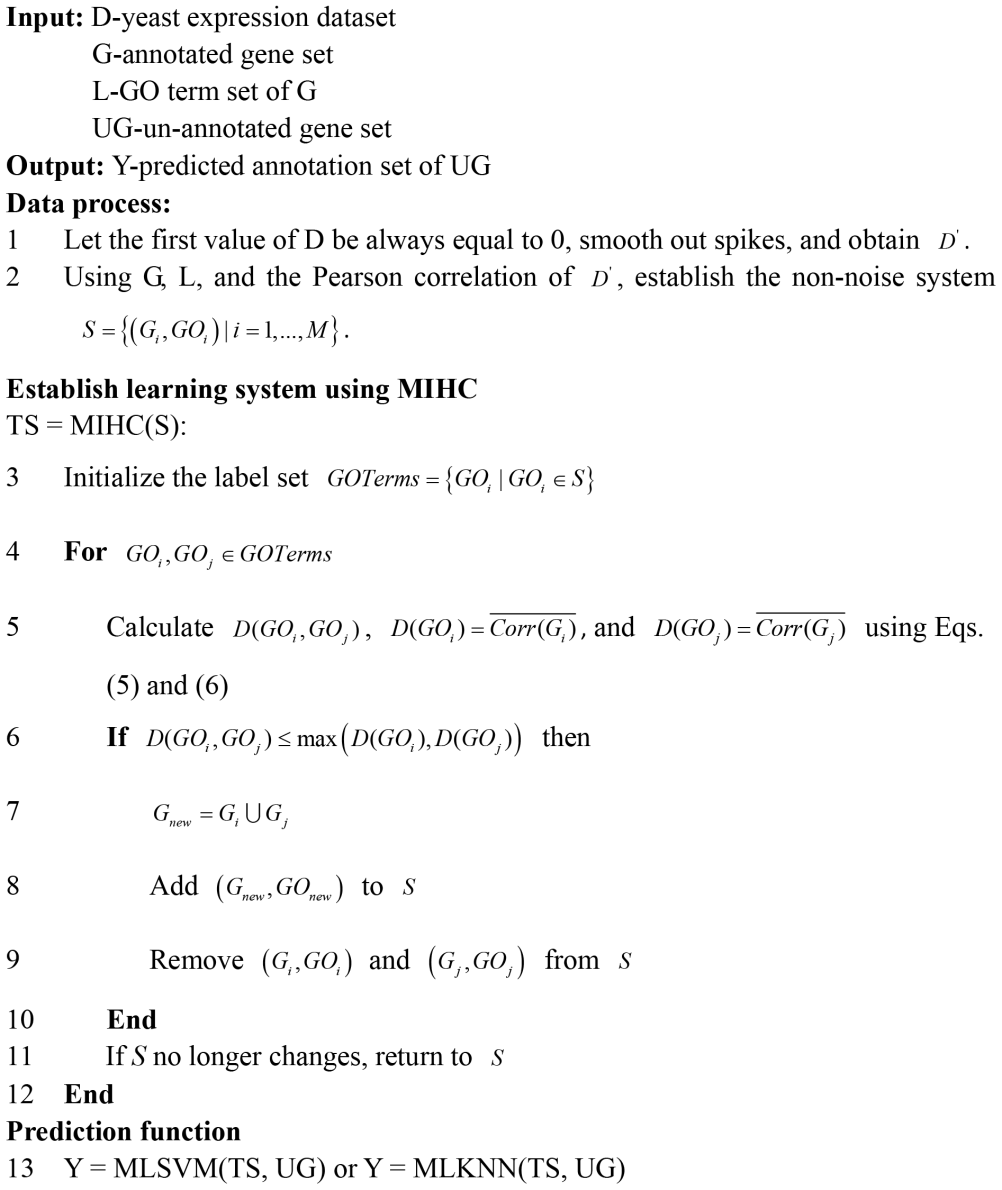
MIHC algorithm and flow chart of function prediction.

To establish a more effective and efficient learning system, we import HCS and propose the novel MIHC method to establish a new learning system with the inherent characteristics of non-noise system 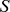 by cluster GO terms. In this method, we treat the relationship 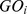 between 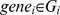 as multi-instance. Our samples (i.e., GO) are different from the traditional HC [28]–[30] because they are multi-instances not instances. Therefore, the distance of each sample is redesigned. According to [31], we define the distance as follows:
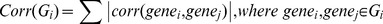
(5)

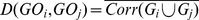
(6)Where 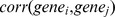 is the Pearson correlation of 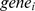 and 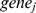. Figure 3 shows the MIHC algorithm and flow chart of function prediction.

## Results and Discussion

### Data

The yeast time-course expression datasets used in this study are obtained from [32] (downloaded from http://genome-www.stanford.edu/cellcycle/data/rawdata/). The four datasets are yeast cell cycle expression data with different time points and circumstances. We use the method in [3], preprocess the raw data, and make the first value always equal to zero. Then, the average transformation 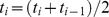 is used to smooth out the spikes. Gene annotation data can be obtained from GO [33] (downloaded from http://www.geneontology.org/GO.downloads.annotations.shtml). GO terms are composed of three disjointed DAGs, namely, biological process (BP), molecular function, and cellular component. We only use BP for this study because it is more complete than the two other disjointed DAGS.

### Performance evaluation

Leave-one-out and leave-a-percent-out cross validation [14] approaches are two of the most extensively used methods for evaluating the performance of a function prediction algorithm. The former is usually used in a small dataset, whereas the latter is more suitable to a large dataset. The former method randomly leaves one sample of the experiment dataset for testing and assigns all of the other samples for training. This process is repeated many times. Meanwhile, the latter method splits the experiment dataset into two sets, namely, the training and testing sets. The training set is composed of a specified proportion of positive and negative samples, whose labels are known. Conversely, the labels of the testing set are concealed from the classifiers. The proportion of the training dataset is gradually increased to test the performance of the learning system. The true labels of the testing set are compared with the prediction labels to evaluate the performance of the system. We select the latter method to evaluate the MIHC method. To accurately measure the performance, the receiver operating characteristic (ROC) curve and area under the ROC curve (AUC) are introduced to quantify the results. The classifications are often based on continuous random variables. The probability of belonging in a class varies with different threshold parameters. That is, the values of true and false positive rates (TPR and FPR, respectively) vary with different threshold parameters. The ROC curve parametrically plots the TPR versus the FPR with varying parameters. The TPR and FPR are calculated by Equations (7) and (8).
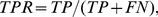
(7)

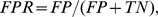
(8)Where TP, FP, TN, FN represent the number of true positive (TP), false positive (FP), true negative (TN), and false negative (FN) predictions, respectively. Therefore, the TPR and FPR can reflect the sensitivity and specificity of prediction. AUC is calculated to quantify the content of the ROC curves. A reliable and valid AUC estimate can be interpreted as the probability that the classifier will assign a higher score to a randomly chosen positive sample rather than to a randomly chosen negative sample.

### Experiment analysis

The four yeast time-course expression datasets are as follows: alpha, cdc15, cdc28, and elution, which record the mRNA levels of 18, 24, 17, and 14 time points in the whole cell cycle under different circumstances, respectively. For each expression dataset, GNC (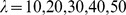), GOLC (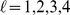), and MIHC methods are used to establish the learning system and compare their performances. The rationale for setting the value of the previously mentioned parameter is as follows. First, we want to determine whether different numbers and different levels of gene group remarkably change function prediction. Second, for the GOLC method, the error rate of a given level is accumulated if a deeper level gene function is required.

The number of genes in the MIHC learning system is consistent with the non-noise system, but other learning systems cannot maintain this feature. Table 1 shows the number of genes and classes for each learning system. The MIHC learning system also has better class features than other learning systems.

**Table 1:** Number of genes and classes in each learning system.

		Gene Number				Class Number			
Method	Parameters	alpha	cdc15	cdc28	elution	alpha	cdc15	cdc28	elution
GNC	λ = 10	1213	1284	224	2089	190	204	27	281
	λ = 20	1216	1294	221	2050	119	129	19	166
	λ = 30	1215	1297	236	2027	84	89	18	122
	λ = 40	1180	1267	204	2022	56	61	10	99
	λ = 50	1205	1207	183	2038	51	42	4	80
GOLC	ι = 1	1334	1417	261	2269	16	15	13	15
	ι = 2	1332	1417	261	2269	49	44	29	51
	ι = 3	1324	1407	261	2263	177	182	85	209
	ι = 4	1278	1341	241	2179	335	342	106	381
MIHC	null	1334	1417	261	2269	74	61	24	29

**Figure 4:**
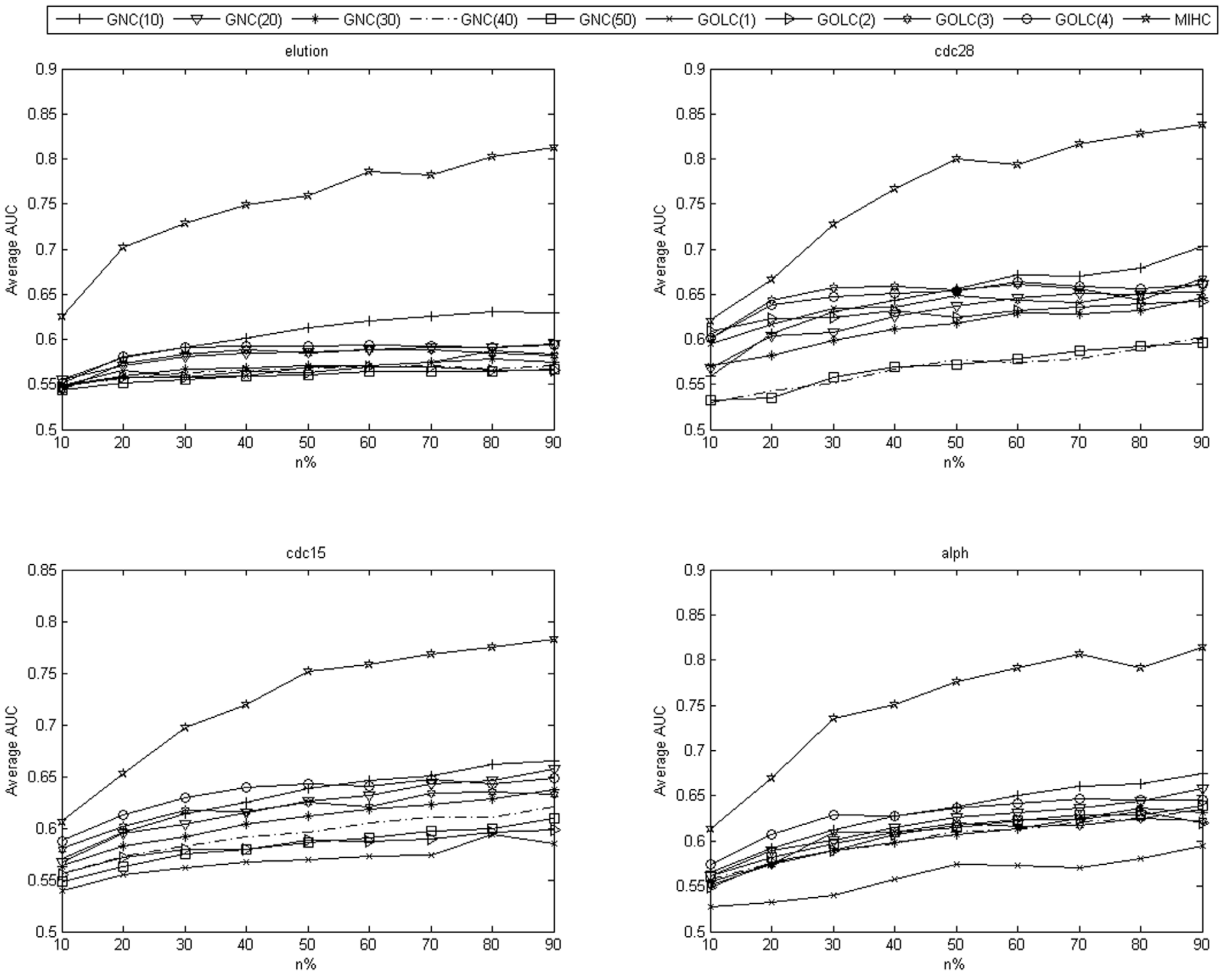
Average AUC obtained from each learning system by MLSVM in all datasets.

**Figure 5:**
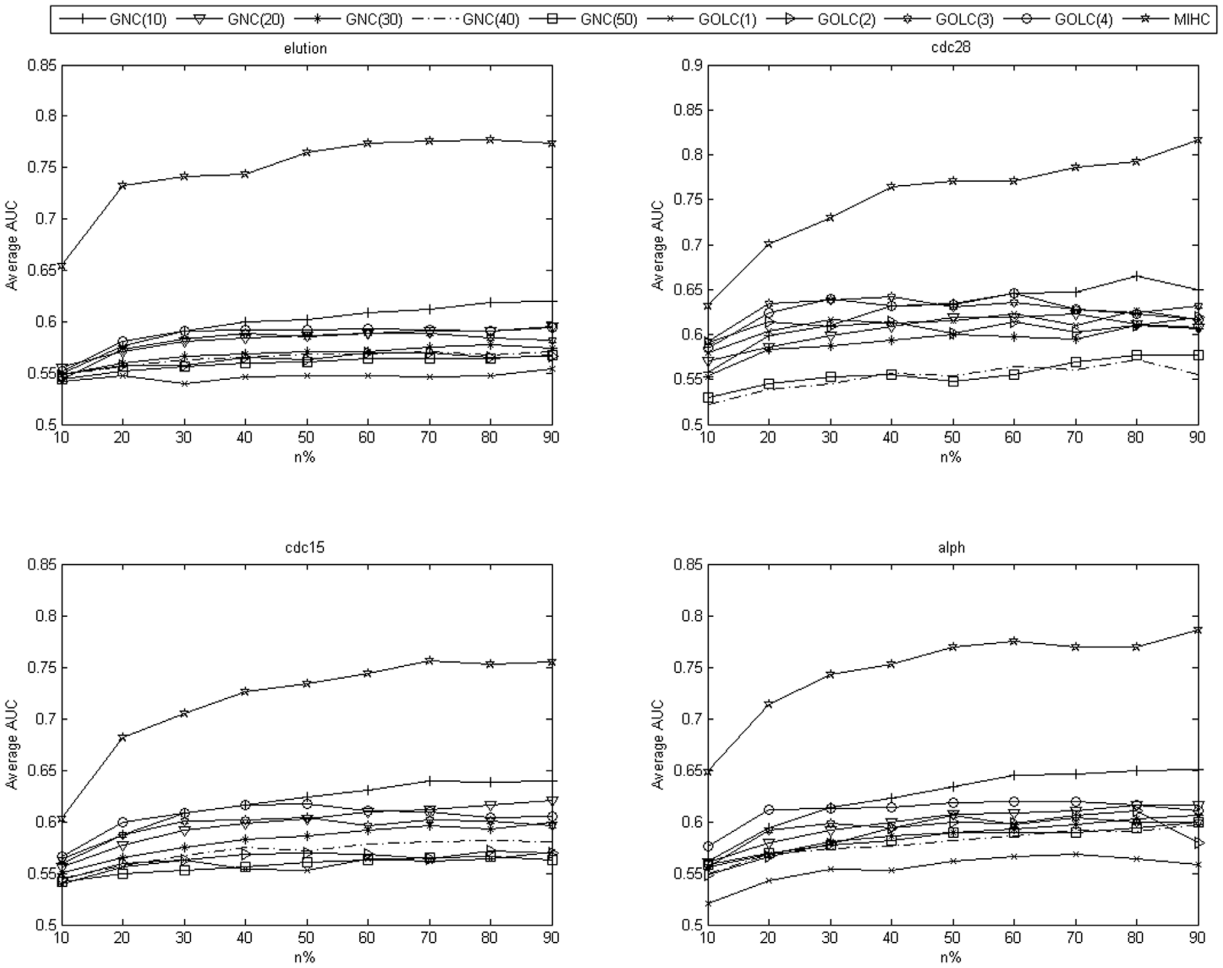
Average AUC obtained from each learning system by MLKNN for each expression dataset.

The MIHC learning system is tested on MLSVM and MLKNN classifiers. In the classification task, the MLL task is decomposed into a series of binary classification tasks. However, the negative samples are far more than the positive samples for each class. Therefore, class imbalance problem should be considered. Further information about the number of positive and negative samples in the cdc28 and elution experiment datasets are shown in Table S1 and Table S2 in the Supporting Information section. The training samples have to be balanced, that is, the same numbers of negative and positive samples are used for the training and testing sets. For each class, we randomly select n% of positive samples and the same number of negative samples as the training set, and the rest are for the testing set. The value of n% increased from 10% to 90%. If the number of positive samples in one class is very low (less than 10), the number of positive samples in the training set is increased gradually. The experiment is repeated 20 times (or more, and the mean value shows minimal changes) for each n, and the mean value is calculated. Given the class imbalance, a high accuracy can still be obtained when the classifier divides all the samples into negative. In this study, AUC is used to evaluate the performance of MIHC. We compare MIHC with GOLC and GNC. For each expression dataset, the average results obtained from each learning system by SVM and KNN classifiers are shown in Figures 4 and 5. Tables 2 and 3 show the results from cdc28 dataset. As the n% increased, the AUC value of MIHC increased drastically whereas those of GOLC and GNC increased slowly. These results prove that generally, the classes in the MIHC learning system are more interesting and the genes therein have more correlation power compared with those in the classes in the two other learning systems. This result can be explained as follows. Genes are transcribed into mRNA and then into proteins. To a certain extent, the level of mRNA can reflect the amount of protein being generated. However, this amount may be influenced by several factors, such as the decomposition of the speed of mRNA and the switching off of proteins. Cells are so efficient that only the necessary proteins are composed. Therefore, variances in gene expression match the active level of biological process. GNC and GOLC cluster GO by up-propagating it along with the GO DAG. Meanwhile, the MIHC method treats the gene expression profile as the feature of GO and clusters GO to ensure superior performance. Moreover, when GO is further up-propagated, the information that reflects the correlation between genes may be lost. Only the GO dataset determines which genes own which GO and whether or not the gene exerts a certain function of the GO in the experiment dataset. However, we assume that genes exert all their GO because the datasets in our study consist of cell cycle expression data. Compared to GNC and GOLC, MIHC relies on statistical correlation. Consequently, MIHC is less concerned about whether or not the gene exerts the function. This problem will be certainly considered in the future study.

**Table 2:** Average AUC obtained from cdc28 dataset by MLSVM.

		Parameter setting for n%								
Method	Parameters	10%	20%	30%	40%	50%	60%	70%	80%	90%
GNC	λ = 10	0.559	0.606	0.631	0.644	0.656	0.671	0.670	0.679	0.703
	λ = 20	0.569	0.603	0.607	0.625	0.638	0.646	0.650	0.650	0.661
	λ = 30	0.571	0.583	0.599	0.612	0.618	0.629	0.628	0.632	0.646
	λ = 40	0.529	0.543	0.552	0.567	0.577	0.574	0.578	0.589	0.602
	λ = 50	0.532	0.535	0.558	0.569	0.572	0.579	0.588	0.592	0.596
GOLC	ι = 1	0.594	0.617	0.634	0.635	0.648	0.643	0.641	0.651	0.653
	ι = 2	0.609	0.623	0.624	0.631	0.624	0.632	0.635	0.640	0.643
	ι = 3	0.601	0.644	0.657	0.658	0.654	0.661	0.656	0.643	0.668
	ι = 4	0.601	0.638	0.647	0.651	0.654	0.663	0.658	0.656	0.662
MIHC	null	**0.621**	**0.666**	**0.727**	**0.767**	**0.800**	**0.794**	**0.817**	**0.828**	**0.838**

**Table 3:** Average AUC obtained from cdc28 dataset by MLKNN.

		Parameter setting for n%								
Method	Parameters	10%	20%	30%	40%	50%	60%	70%	80%	90%
GNC	λ = 10	0.550	0.576	0.591	0.599	0.603	0.609	0.619	0.619	0.620
	λ = 20	0.556	0.571	0.581	0.585	0.586	0.589	0.591	0.591	0.595
	λ = 30	0.548	0.559	0.567	0.568	0.571	0.571	0.575	0.578	0.575
	λ = 40	0.548	0.557	0.563	0.565	0.569	0.569	0.571	0.567	0.571
	λ = 50	0.544	0.552	0.556	0.559	0.560	0.564	0.564	0.564	0.567
GOLC	ι = 1	0.541	0.547	0.540	0.546	0.547	0.547	0.547	0.547	0.554
	ι = 2	0.548	0.557	0.558	0.565	0.563	0.569	0.569	0.566	0.566
	ι = 3	0.544	0.573	0.584	0.588	0.585	0.589	0.589	0.585	0.582
	ι = 4	0.552	0.580	0.591	0.592	0.592	0.594	0.592	0.591	0.594
MIHC	null	**0.655**	**0.733**	**0.741**	**0.743**	**0.765**	**0.774**	**0.776**	**0.777**	**0.774**

**Figure 6:**
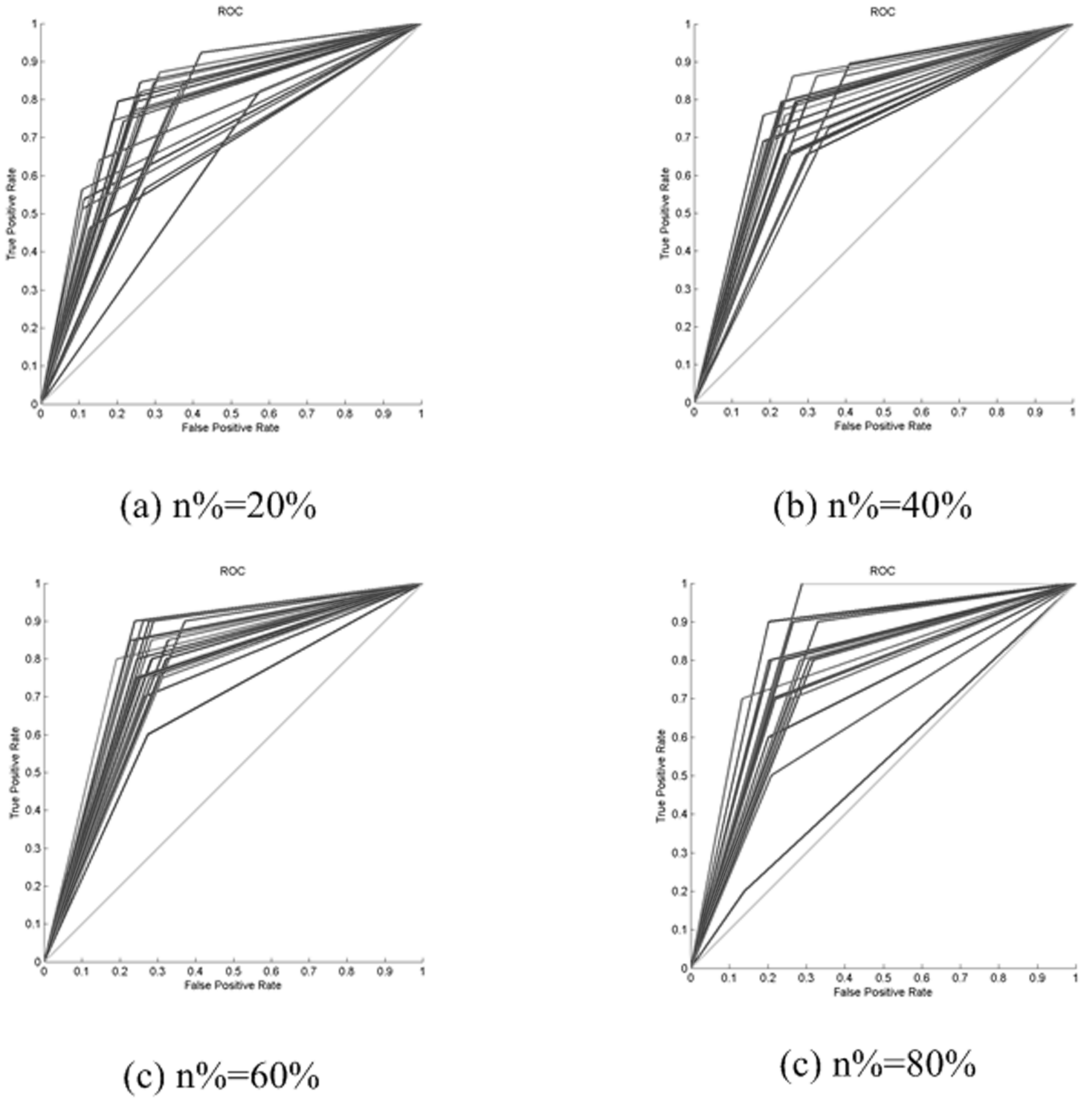
The ROC of a class obtained from the MIHC learning system by MLSVM for the cdc28 dataset. The ROC curves of the 20 repetitions of the experiment as well as the four subplots (a), (b), (c), and (d) with parameter n% = 20%, 40%, 60%, and 80%, respectively, are shown.

Lastly, to obtain a satisfactory explanation in a real-world problem, the ROC curve of a class obtained from the MIHC learning system for the cdc28 dataset is shown in Figure 6. The results for the TP, FP, TN, and FN are shown in Table 4 (given that the experiment is repeated 20 times, only the middle-level results for the 2 repetitions are shown in Table 4; the average TPR and FPR in the 20 repetitions of the experiment are shown in Table 5). In Figure 6, the ROC curves of the 20 repetitions of the experiment as well as the four subplots (a), (b), (c), and (d) with parameter n% = 20%, 40%, 60%, and 80%, respectively, are displayed. As n increases, the number of positive samples in the testing set decreases. The classifier sometimes pays a greater price to identify as many positive samples in the testing set as possible. The sample distribution in training set may also influence the prediction result. The ROC curve in subplot (d) occasionally exhibits unsatisfactory performance. The ROC curves of all the datasets are presented in Figures S1 to S8, and the average TPR and FPR of all the datasets are shown in Tables S3 to S10 in the Supporting Information section.

**Table 4:** Results of the TP, FP, TN, and FN in MIHC by MLSVM.

				First experiment results				Second experiment results			
n%	Train/Test	+	−	TP	FP	TN	FN	TP	FP	TN	FN
10%	Train	5	5								
	Test	44	207	19	45	162	25	22	30	177	22
20%	Train	10	10								
	Test	39	202	25	31	171	14	29	38	164	10
30%	Train	15	15								
	Test	34	197	24	40	157	10	26	44	153	8
40%	Train	20	20								
	Test	29	192	23	51	141	6	21	68	124	8
50%	Train	25	25								
	Test	24	187	17	54	133	7	20	55	132	4
60%	Train	29	29								
	Test	20	183	17	44	139	3	15	44	139	5
70%	Train	34	34								
	Test	15	178	11	41	137	4	12	50	128	3
80%	Train	39	39								
	Test	10	173	7	37	136	3	8	36	137	2
90%	Train	44	44								
	Test	5	168	4	40	128	1	5	36	132	0

1. ‘+’ means positive sample number; ‘−’ means negative sample number.

**Table 5:** Average TPR and FPR in MIHC by MLSVM.

n%	TPR	FPR
10%	0.713	0.286
20%	0.733	0.255
30%	0.760	0.250
40%	0.757	0.264
50%	0.783	0.257
60%	0.808	0.272
70%	0.783	0.241
80%	0.745	0.234
90%	0.810	0.243

## Conclusion

In this paper, we propose the MIHC method to establish a learning system, which is verified by SVM and KNN using four yeast gene expression datasets. In the MIHC method, Pearson correlation is the distance between multi-instance samples, and HC is used to cluster the samples. Compared with other learning system establishment methods, the MIHC learning system exhibits better performance because the samples are more easily recognized. This method also maintains data integrity with non-noise system. To our knowledge, this study is the first to use HC algorithm to cluster multi-instance samples.

## Supporting Information

Figure S1 **ROC curves are obtained from cdc28 dataset by MLSVM.** The ROC curves of each learning system, generated by average TPR and FPR, as well as the four subplots (a), (b), (c), and (d) with parameter n% = 20%, 40%, 60%, and 80%, respectively, are shown.
(TIF)

Figure S2 **ROC curves are obtained from cdc28 dataset by MLKNN.** The ROC curves of each learning system, generated by average TPR and FPR, as well as the four subplots (a), (b), (c), and (d) with parameter n% = 20%, 40%, 60%, and 80%, respectively, are presented.
(TIF)

Figure S3 **ROC curves are obtained from cdc15 dataset by MLSVM.** The ROC curves of each learning system, generated by average TPR and FPR, as well as the four subplots (a), (b), (c), and (d) with parameter n% = 20%, 40%, 60%, and 80%, respectively, are shown.
(TIF)

Figure S4 **ROC curves are obtained from cdc15 dataset by MLKNN.** The ROC curves of each learning system, generated by average TPR and FPR, as well as the four subplots (a), (b), (c), and (d) with parameter n% = 20%, 40%, 60%, and 80%, respectively, are displayed.
(TIF)

Figure S5 **ROC curves are obtained from alpha dataset by MLSVM.** The ROC curves of each learning system, generated by average TPR and FPR, as well as the four subplots (a), (b), (c), and (d) with parameter n% = 20%, 40%, 60%, and 80%, respectively, are displayed.
(TIF)

Figure S6 **ROC curves are obtained from alpha dataset by MLKNN.** The ROC curves of each learning system, generated by average TPR and FPR, as well as the four subplots (a), (b), (c), and (d) with parameter n% = 20%, 40%, 60%, and 80%, respectively, are presented.
(TIF)

Figure S7 **ROC curves are obtained from elution dataset by MLSVM.** The ROC curves of each learning system, generated by average TPR and FPR, as well as the four subplots (a), (b), (c), and (d) with parameter n% = 20%, 40%, 60%, and 80%, respectively, are shown.
(TIF)

Figure S8 **ROC curves are obtained from elution dataset by MLKNN.** The ROC curves of each learning system, generated by average TPR and FPR, as well as the four subplots (a), (b), (c), and (d) with parameter n% = 20%, 40%, 60%, and 80%, respectively, are displayed.
(TIF)

Table S1 **Number of positive and negative samples in MIHC from the cdc28 dataset.**
(XLS)

Table S2 **Number of positive and negative samples in MIHC from the elution dataset.**
(XLS)

Table S3 **Average TPR and FPR obtained from the cdc28 dataset by MLKNN.**
(XLS)

Table S4 **Average TPR and FPR obtained from the cdc28 dataset by MLSVM.**
(XLS)

Table S5 **Average TPR and FPR obtained from the cdc15 dataset by MLKNN.**
(XLS)

Table S6 **Average TPR and FPR obtained from the cdc15 dataset by MLSVM.**
(XLS)

Table S7 **Average TPR and FPR obtained from the alpha dataset by MLKNN.**
(XLS)

Table S8 **Average TPR and FPR obtained from the alpha dataset by MLSVM.**
(XLS)

Table S9 **Average TPR and FPR obtained from the elution dataset by MLKNN.**
(XLS)

Table S10 **Average TPR and FPR obtained from the elution dataset by MLSVM.**
(XLS)
