# Supplementary figures and images for: Using Multi-Instance Hierarchical Clustering Learning System to Predict Yeast Gene Function
Source: PLoS One. 2014 Mar 12;9(3):e90962. doi: 10.1371/journal.pone.0090962 (PMC3951281; doi:10.1371/journal.pone.0090962)

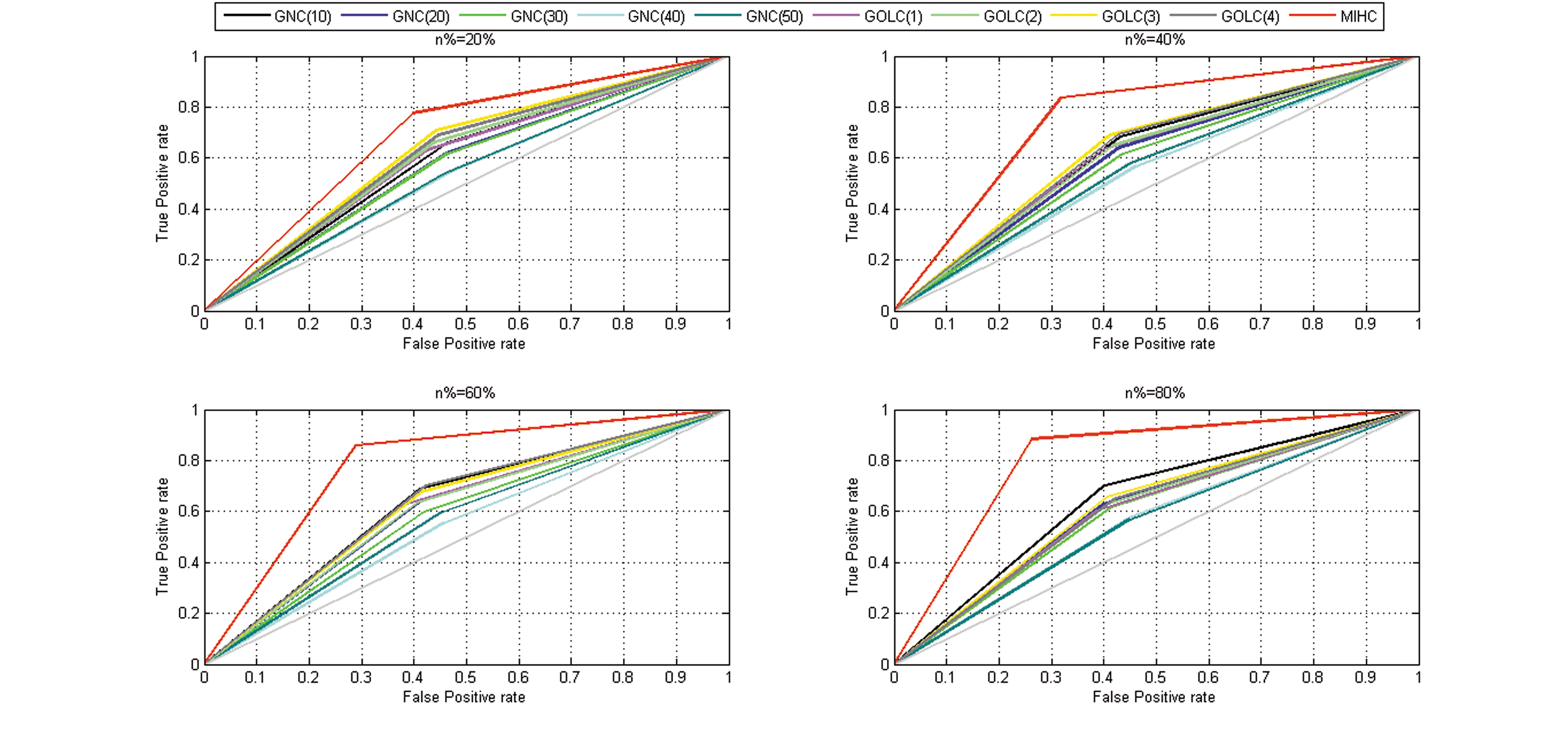

Supplement: Figure S1 — ROC curves are obtained from cdc28 dataset by MLSVM. The ROC curves of each learning system, generated by average TPR and FPR, as well as the four subplots (a), (b), (c), and (d) with parameter n% = 20%, 40%, 60%, and 80%, respectively, are shown. (TIF) [file pone.0090962.s001.tif]

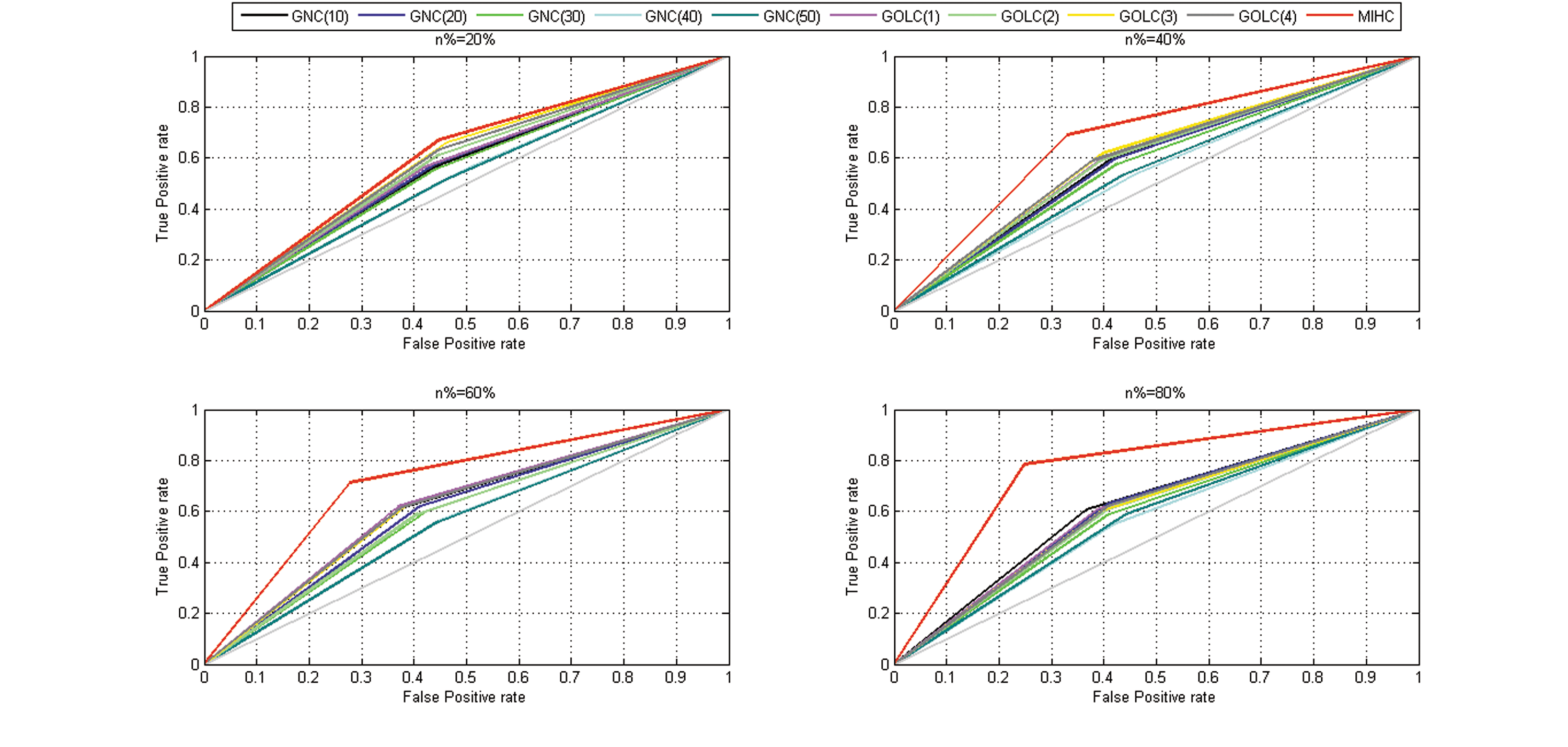

Supplement: Figure S2 — ROC curves are obtained from cdc28 dataset by MLKNN. The ROC curves of each learning system, generated by average TPR and FPR, as well as the four subplots (a), (b), (c), and (d) with parameter n% = 20%, 40%, 60%, and 80%, respectively, are presented. (TIF) [file pone.0090962.s002.tif]

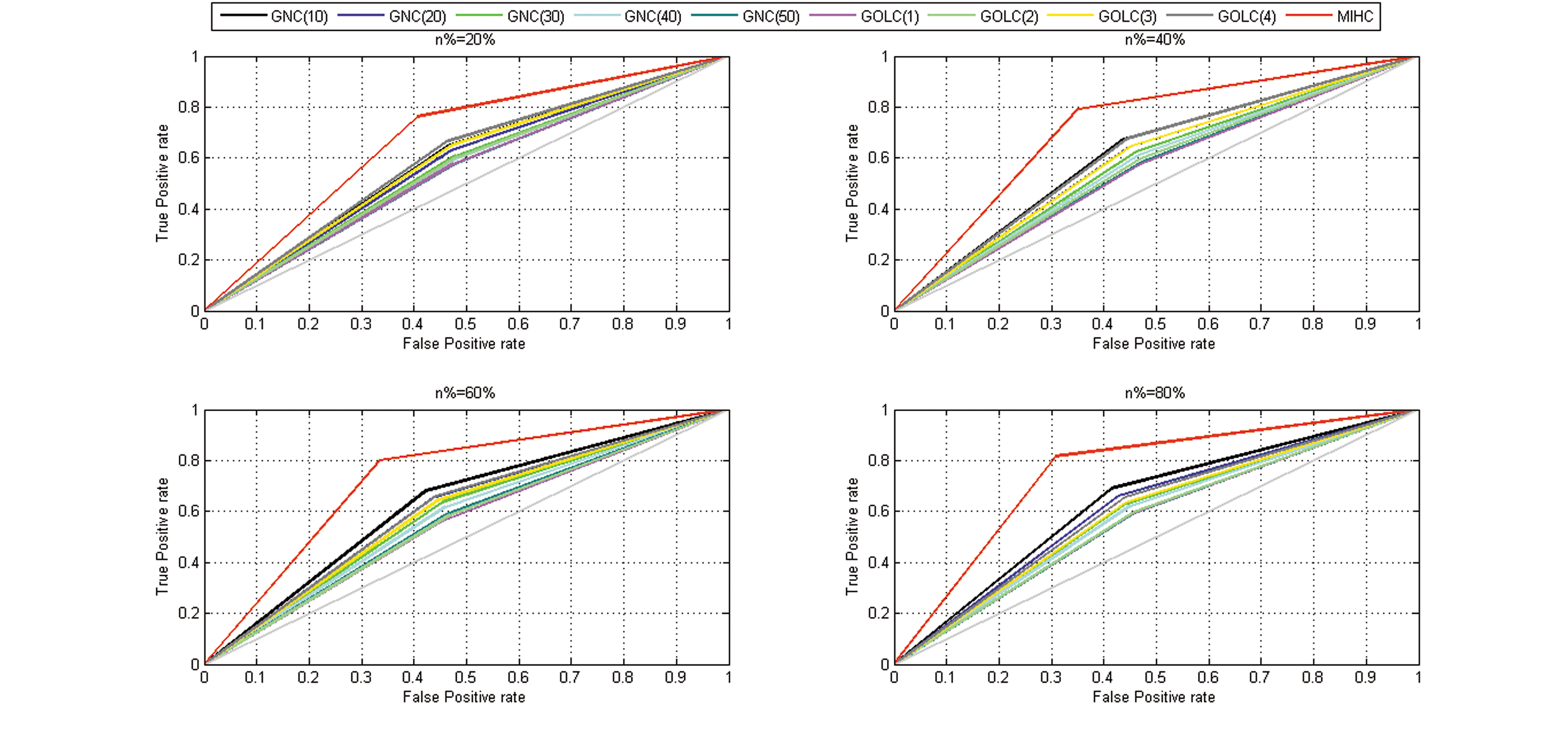

Supplement: Figure S3 — ROC curves are obtained from cdc15 dataset by MLSVM. The ROC curves of each learning system, generated by average TPR and FPR, as well as the four subplots (a), (b), (c), and (d) with parameter n% = 20%, 40%, 60%, and 80%, respectively, are shown. (TIF) [file pone.0090962.s003.tif]

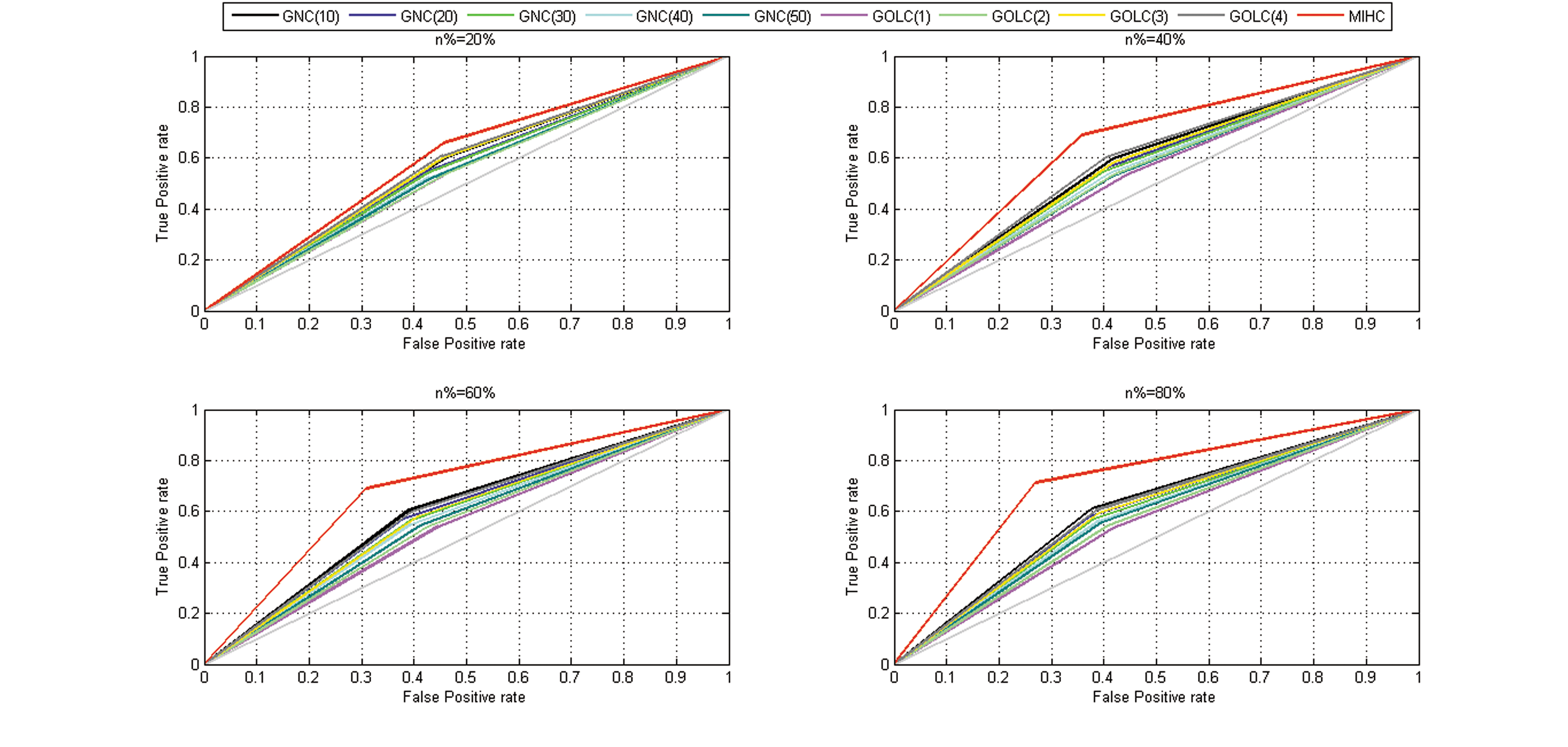

Supplement: Figure S4 — ROC curves are obtained from cdc15 dataset by MLKNN. The ROC curves of each learning system, generated by average TPR and FPR, as well as the four subplots (a), (b), (c), and (d) with parameter n% = 20%, 40%, 60%, and 80%, respectively, are displayed. (TIF) [file pone.0090962.s004.tif]

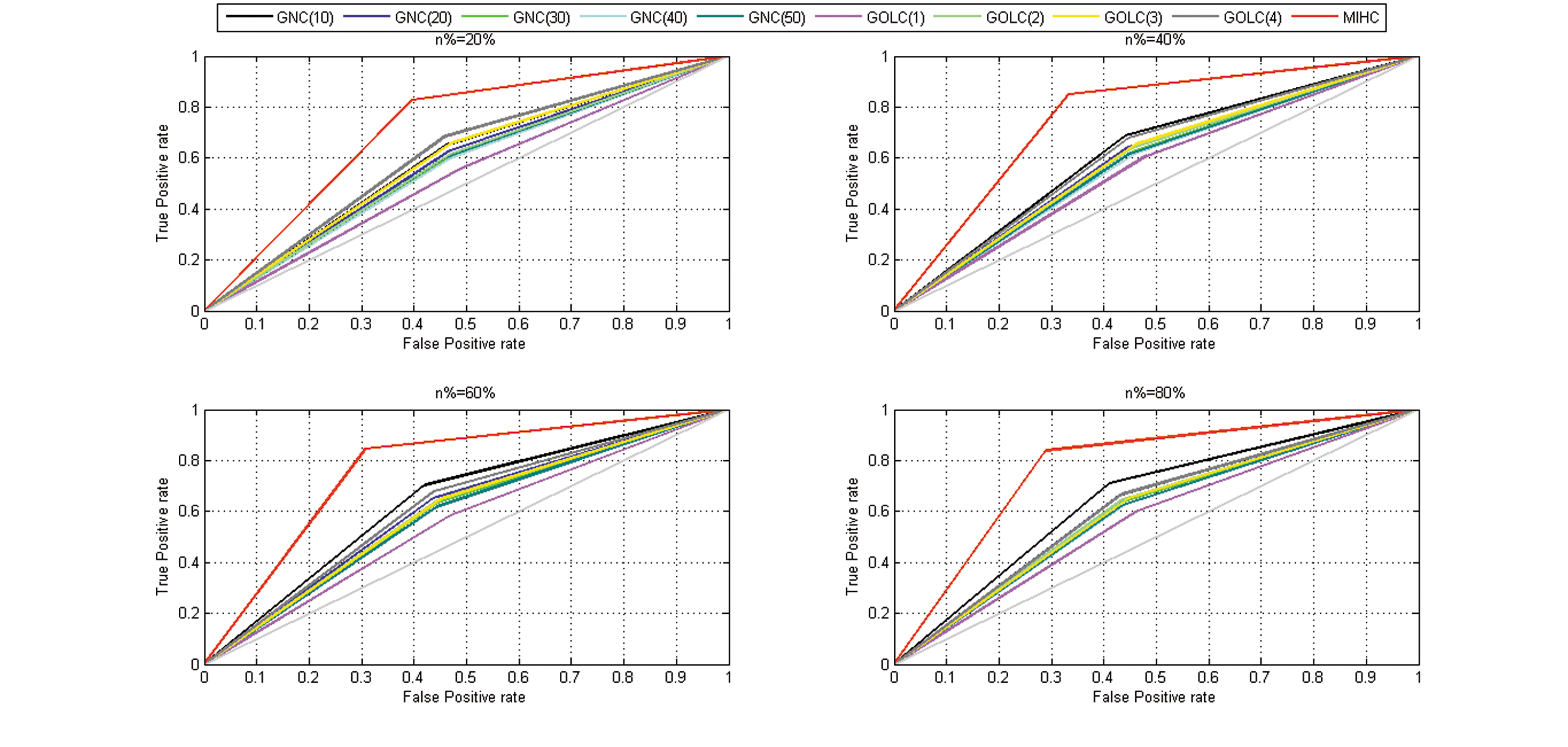

Supplement: Figure S5 — ROC curves are obtained from alpha dataset by MLSVM. The ROC curves of each learning system, generated by average TPR and FPR, as well as the four subplots (a), (b), (c), and (d) with parameter n% = 20%, 40%, 60%, and 80%, respectively, are displayed. (TIF) [file pone.0090962.s005.tif]

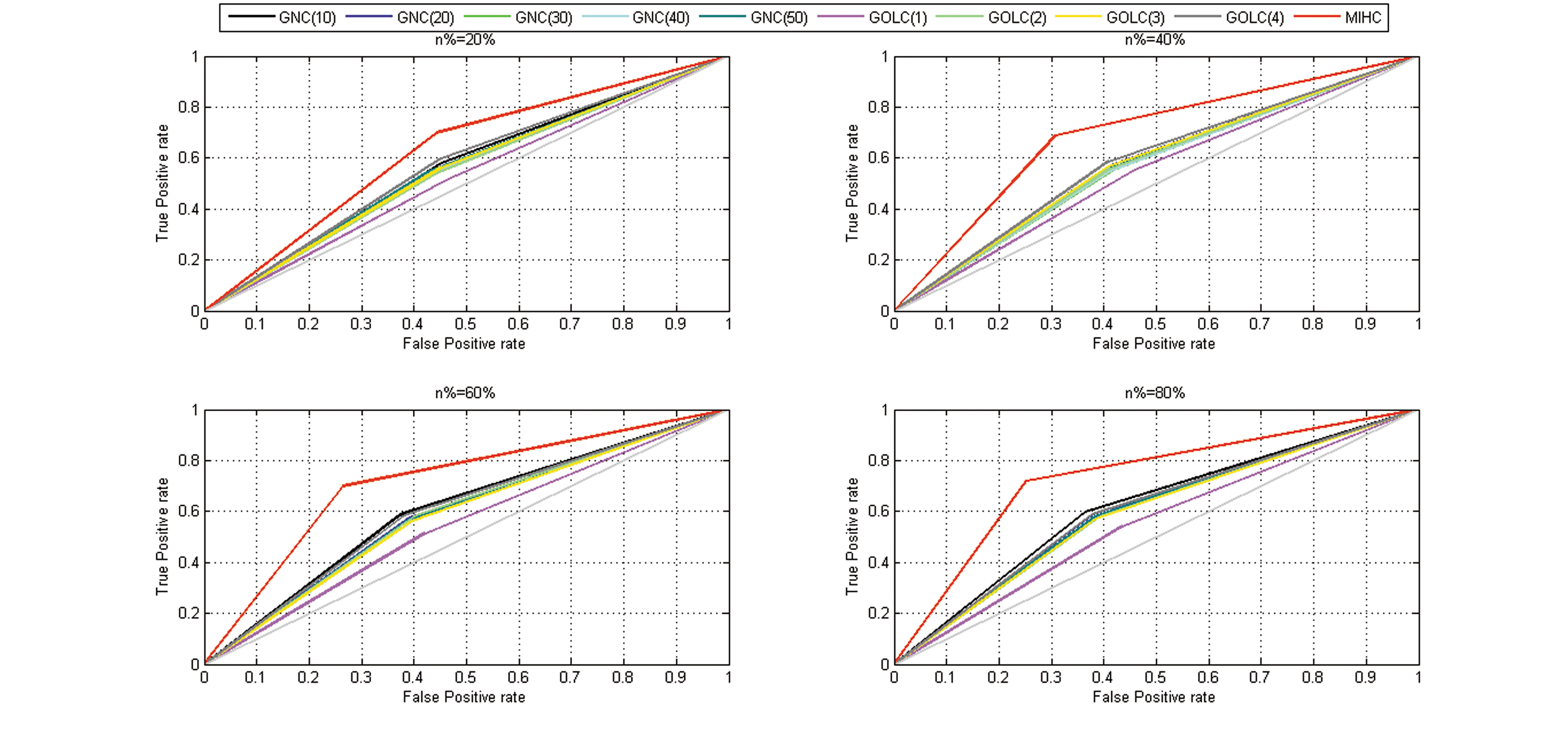

Supplement: Figure S6 — ROC curves are obtained from alpha dataset by MLKNN. The ROC curves of each learning system, generated by average TPR and FPR, as well as the four subplots (a), (b), (c), and (d) with parameter n% = 20%, 40%, 60%, and 80%, respectively, are presented. (TIF) [file pone.0090962.s006.tif]

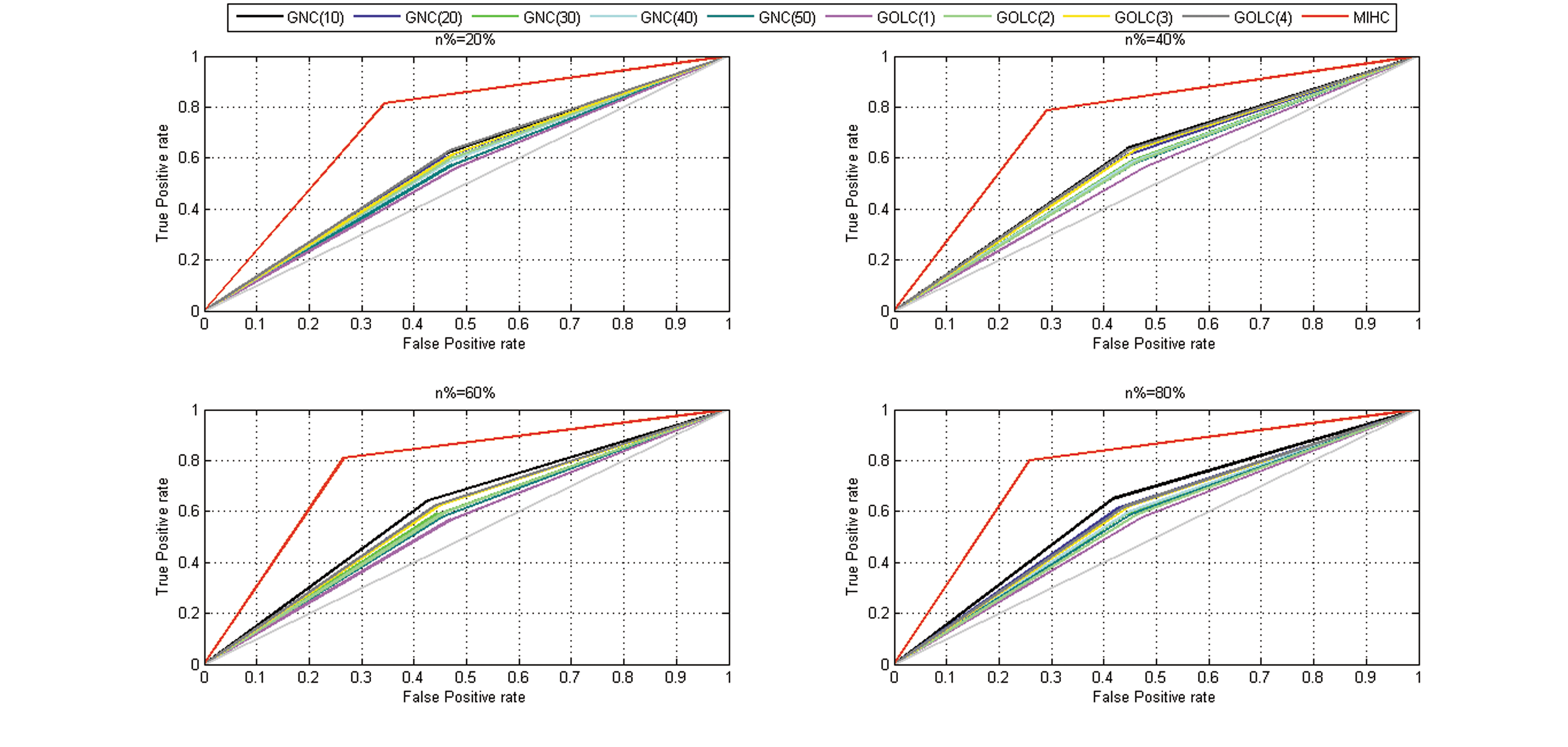

Supplement: Figure S7 — ROC curves are obtained from elution dataset by MLSVM. The ROC curves of each learning system, generated by average TPR and FPR, as well as the four subplots (a), (b), (c), and (d) with parameter n% = 20%, 40%, 60%, and 80%, respectively, are shown. (TIF) [file pone.0090962.s007.tif]

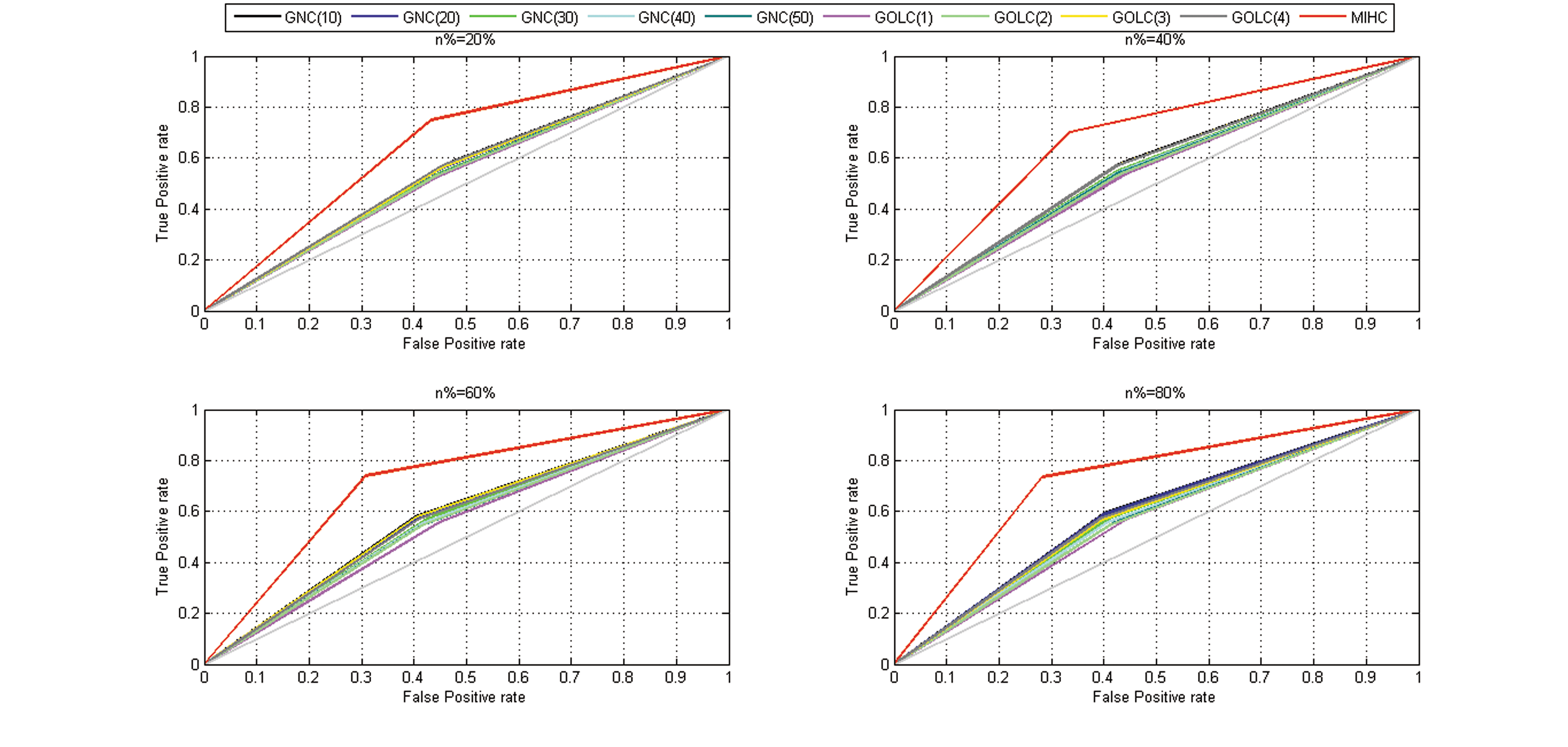

Supplement: Figure S8 — ROC curves are obtained from elution dataset by MLKNN. The ROC curves of each learning system, generated by average TPR and FPR, as well as the four subplots (a), (b), (c), and (d) with parameter n% = 20%, 40%, 60%, and 80%, respectively, are displayed. (TIF) [file pone.0090962.s008.tif]
